# Endovascular Thrombectomy for Radial Artery Occlusion in Neuro-Interventional Procedures: A Report of Two Cases

**DOI:** 10.7759/cureus.53337

**Published:** 2024-01-31

**Authors:** Travis Atchley, Philip Schmalz

**Affiliations:** 1 Neurological Surgery, University of Alabama at Birmingham, Birmingham, USA

**Keywords:** mechanical thrombectomy, case report, radial artery access, radial artery occlusion, endovascular thrombectomy

## Abstract

Transradial access has garnered increasing popularity and acceptance among the neuro-interventional community. As this technique becomes commonplace for both diagnostic and interventional procedures, an understanding of potential complications and management is tantamount. Here, we describe two cases of thrombosed radial arteries successfully recanalized with traditional thrombectomy techniques.

Two patients presented with aneurysmal subarachnoid hemorrhage and were found to have ruptured anterior communicating artery aneurysms. Both patients were deemed appropriate candidates for endovascular treatment. During attempted access, both patients were found to have occluded right radial arteries due to previous arterial access, one from a prior intervention and one from an arterial line placed at another facility. Thrombectomies were subsequently performed, one via manual aspiration through the access sheath and the other with a commercially available aspiration system. Both radial arteries were successfully recanalized and the interventions were completed via transradial access.

Endovascular thrombectomy for radial artery thrombosis is a feasible and simple technique that can be employed to facilitate transradial access for neuro-endovascular procedures when a thrombosed radial artery is encountered. This technique can be attempted in cases of radial artery thrombosis prior to conversion to transfemoral access.

## Introduction

Transradial access (TRA) has gained increasing popularity for neuro-interventional procedures. TRA has reported a lower rate of access site complications compared to traditional transfemoral access (TFA) [1-2]. There is also an overall trend toward better patient satisfaction with TRA versus TFA [2]. However, TRA is not without complications and technical challenges, of which the neuro-interventionalist must be cognizant. Radial artery spasm, arterial occlusion, and hematoma are the most commonly cited complications and may occur in as often as 10% of cases [1]. However, when choosing an access route, it’s also necessary to recognize situations that may preclude TRA. Patients with unfavorable collateral circulation as assessed by the Allen’s or Barbeau tests are not suitable [3-4]. Patients with suspected radial artery occlusion (RAO) were not deemed candidates for TRA and usually underwent TFA instead. Radial artery thrombus/occlusion after TRA occurs in 5-10% of cases, but the true incidence of RAO before TRA is likely underreported [2-3,5]. Here, we report the novel application of mechanical thrombectomy techniques in two cases to re-open an occluded radial artery with excellent response and no adverse events.

## Case presentation

**Figure 1:**
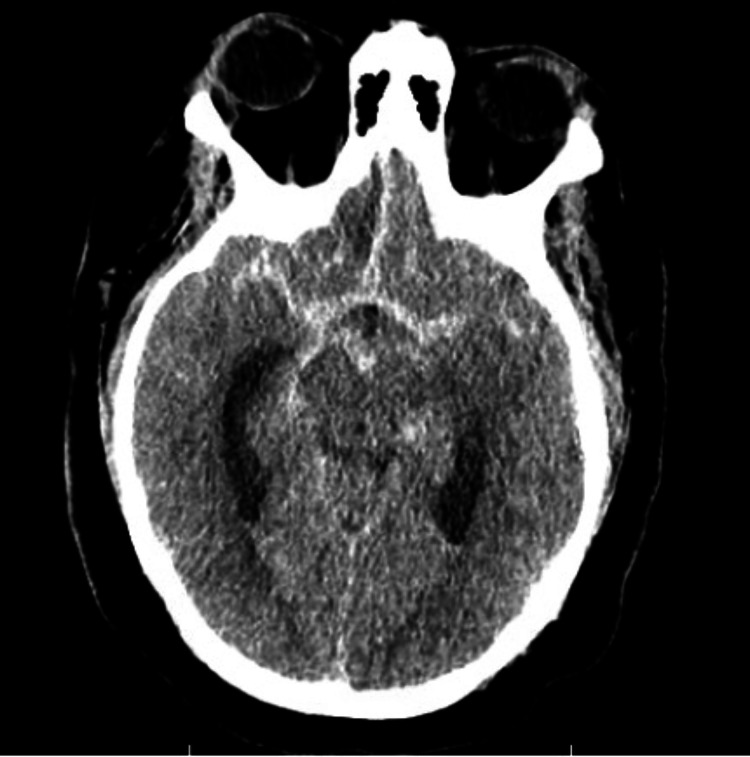
Non-contrast computed tomography scan with diffuse cisternal subarachnoid hemorrhage extending into the interhemispheric fissure with associated obstructive hydrocephalus

**Figure 2:**
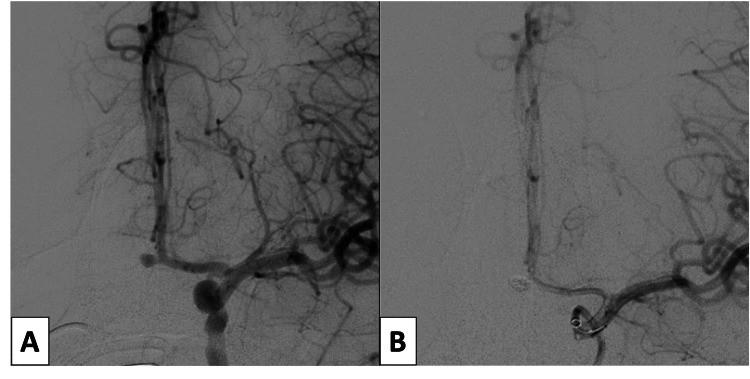
Anterior-posterior view of the left internal carotid artery injection demonstrating a saccular anterior communicating complex aneurysm before (A) and after successful coil embolization (B)

The first case was of a 42-year-old female with a Hunt and Hess (HH) grade IV aneurysmal subarachnoid hemorrhage (SAH) (Figure 1) [6]. Noninvasive imaging suggested an anterior communicating artery aneurysm. A right radial arterial line was placed at an outside facility prior to transfer to our hospital. This was re-sited to the left radial artery in anticipation of a planned neuro-endovascular procedure. The following day, after obtaining informed consent, she was taken to the neuro-interventional suite for definitive diagnosis and treatment. Micropuncture access to the radial artery was established, and a 6 French (Fr) sheath was inserted. Poor backbleeding from the sheath was noted and RAO from the previous arterial line was suspected and subsequently confirmed with radial angiography through the sheath. A Penumbra RED 072 aspiration catheter (Penumbra Inc, Alameda CA) was connected to a heparinized saline flush and advanced through the sheath into the distal brachial artery over an 035 Glidewire. The Penumbra aspiration system (Penumbra Inc, Alameda CA) was used to apply suction to the hub of the aspiration catheter, which was then slowly withdrawn from the sheath. A substantial portion of the clot was recovered, and a follow-up arteriogram demonstrated recanalization of the radial artery. The patient then successfully underwent coil embolization of her aneurysm without complication (Figure 2).

**Figure 3:**
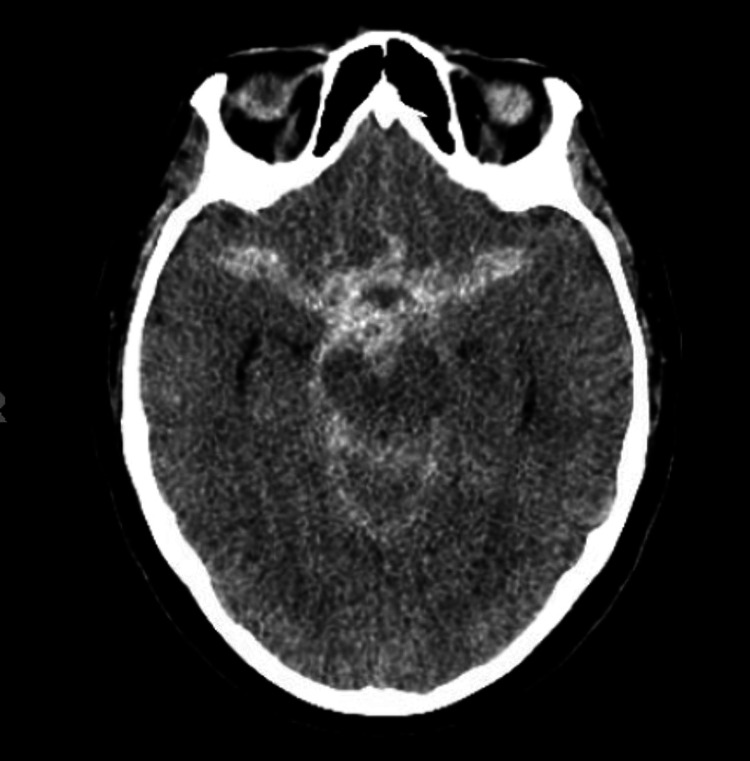
Non-contrast computed tomography scan with a diffuse cisternal subarachnoid hemorrhage

**Figure 4:**
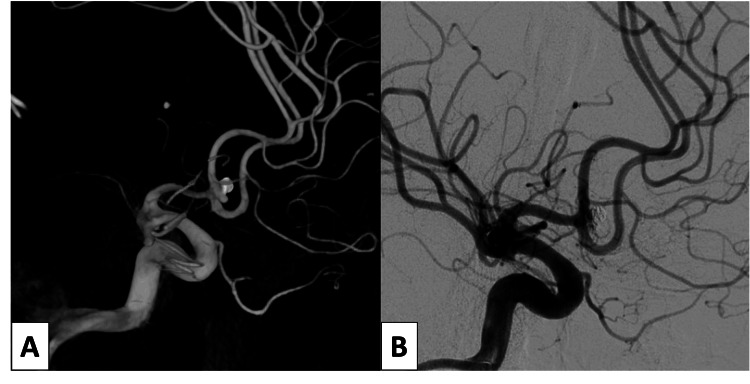
Three-dimensional lateral view of the right internal carotid artery injection demonstrating an anterior communicating complex aneurysm following initial coil embolization (A) and subtracted lateral angiogram following successful stent-assisted coil embolization of complex aneurysm (B)

The second case was of a 72-year-old female who presented with an HH grade III aneurysmal SAH also due to an anterior communicating artery aneurysm (Figure 3). After informed consent was obtained, initial angiography and primary coil embolization of this aneurysm were performed via right-sided TRA without issue. A daughter sac originating from the neck was noted on her interventional angiogram and stent-assisted coiling was planned at a second procedure after initiation of dual antiplatelet therapy. After informed consent, she was taken back to the neuro-interventional suite three days later for stent-assisted coil embolization. Collateral circulation was assessed, and patency of the right radial artery was confirmed using color-flow Doppler. Micropuncture access was established with ultrasound guidance and a 7 Fr sheath was inserted. Despite appropriate sheath placement and adjustment of the sheath position, no return of arterial blood was noted. Given the recent TRA for coil embolization, RAO was suspected. A mechanical thrombectomy was planned. While applying manual negative pressure, the sheath was withdrawn slowly. A significant thrombus was noted within the sheath but no arterial backbleeding was noted. The sheath was then replaced and again withdrawn under manual negative pressure. After this “second pass”, free return of blood was noted. A second 7 Fr sheath was placed, patency confirmed, and stent-assisted coiling was completed without complication (Figure 4). 

## Discussion

**Figure 5:**
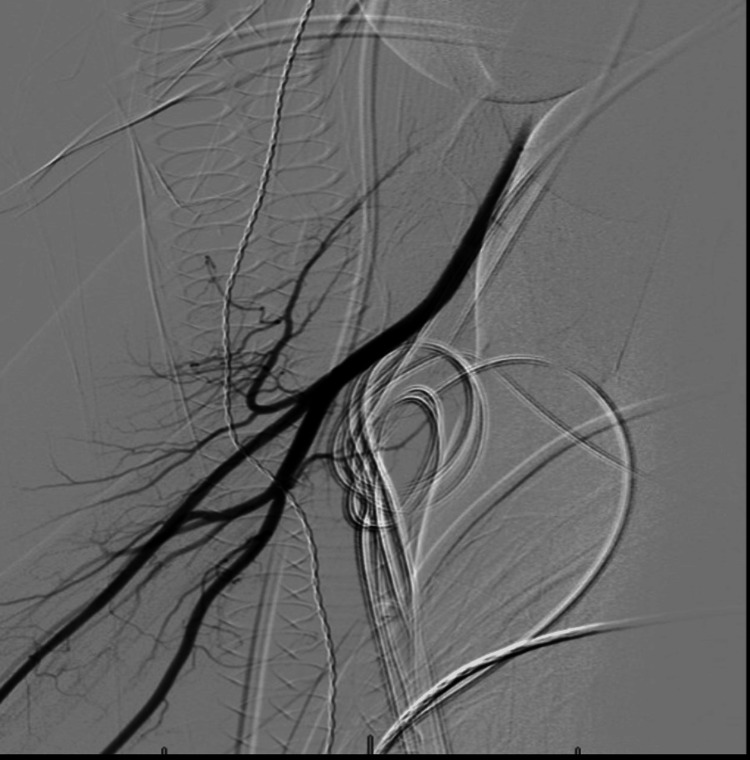
Representative anterior-posterior view of the successfully cannulated right radial artery

Given the increasing popularity of TRA, an understanding of complications and potential troubleshooting techniques is paramount. The TRA risk profile is well-reported, with the most common complications being arterial spasm, thrombus with or without occlusion, site hematoma, perforation, dissection, and pseudoaneurysm [1-2,7]. Two randomized control trials in the interventional cardiology literature have demonstrated safety and comparable outcomes for distal TRA access at the wrist or anatomic snuffbox [8,9]. However, there remains a paucity of literature on potential troubleshooting methods for occluded radial arteries prior to performing neuro-interventional procedures. There have been limited reports of distal radial arterial access for subsequent balloon angioplasty and tissue plasminogen activator (tPA) infusion for an occluded radial artery [10]. Commonly, alternative arterial sites are chosen in the setting of occluded radial arteries such as the contralateral radial artery, femoral arteries, or ulnar arteries [1]. It is also important to note that the radial artery can thrombose proximally while maintaining distal patency. This may be due to patent ulnar artery collaterals providing continuous flow into the distal radial artery branches, thereby precluding distal thrombus formation [11]. During neurointerventional procedures, this may manifest as a patent distal radial artery (either via direct puncture or as evidence on Doppler ultrasonography) with subsequent absent or reduced backbleeding following access sheath placement. This was the case in both described cases. In cases where the radial artery is found to be occluded on Doppler ultrasonography prior to cannulation, seeking an alternative access site should be considered. However, in these cases, the radial artery was patent distally and only found to be occluded after sheath placement. Following thrombectomy, successful radial artery cannulization was obtained in both cases (Figure 5).

We have presented two cases of mechanical thrombectomy for occluded radial arteries to allow TRA for neuro-interventional procedures. To our knowledge, these are the first reported cases employing this technique. Other alternative methods to re-open occluded radial arteries include infusion of heparinized saline or the use of tPA; however, these may not be as efficacious as thrombectomy. Additionally, in the setting of an unsecured, ruptured cerebral aneurysm, the use of potent or high-dose thrombolytics is met with reasonable trepidation. In our cases, we used manual techniques and a commercially available aspiration system for the thrombectomy, both of which were successful. This may suggest that in institutions where resources may be lacking, manual application of negative pressure to an arterial sheath can prove efficacious. Neither of these patients experienced any adverse events related to their radial artery thrombectomy and both were able to subsequently undergo the planned neuro-interventional procedures through the original right radial artery access site. While the procedure would still have been possible either through traditional access routes, this technique minimized additional morbidity to the patient and prevented the risks associated with TFA or ulnar cannulation in the setting of RAO. Thus, we propose the application of either of these radial artery mechanical thrombectomy techniques in cases of RAO prior to changing access sites or infusion of potent thrombolytics, especially in cases of intracranial hemorrhage.

## Conclusions

We have described a simple technique for endovascular thrombectomy of occluded radial arteries in order to perform neuro-interventional procedures in two cases. This procedure employs readily available resources and common neuro-interventional techniques. Radial artery aspiration thrombectomy should be considered prior to abandoning TRA or infusion of thrombolytic therapy. This technique may reduce the potential morbidity of seeking alternative access sites after cannulation of a proximally occluded radial artery.
